# Model of Chronic Equine Endometritis Involving a Pseudomonas aeruginosa Biofilm

**DOI:** 10.1128/IAI.00332-17

**Published:** 2017-11-17

**Authors:** Ryan A. Ferris, Patrick M. McCue, Grace I. Borlee, Kristina E. Glapa, Kevin H. Martin, Mihnea R. Mangalea, Margo L. Hennet, Lisa M. Wolfe, Corey D. Broeckling, Bradley R. Borlee

**Affiliations:** a Department of Clinical Sciences, Colorado State University, Fort Collins, Colorado, USA; b Department of Microbiology, Immunology and Pathology, Colorado State University, Fort Collins, Colorado, USA; c Proteomics and Metabolomics Facility, Colorado State University, Fort Collins, Colorado, USA; University of Massachusetts Medical School

**Keywords:** equine, endometritis, bacteria, biofilm, cyclic di-GMP, exopolysaccharide

## Abstract

Bacteria in a biofilm community have increased tolerance to antimicrobial therapy. To characterize the role of biofilms in equine endometritis, six mares were inoculated with *lux*-engineered Pseudomonas aeruginosa strains isolated from equine uterine infections. Following establishment of infection, the horses were euthanized and the endometrial surfaces were imaged for luminescence to localize adherent *lux*-labeled bacteria. Samples from the endometrium were collected for cytology, histopathology, carbohydrate analysis, and expression of inflammatory cytokine genes. Tissue-adherent bacteria were present in focal areas between endometrial folds (6/6 mares). The Pel exopolysaccharide (biofilm matrix component) and cyclic di-GMP (biofilm-regulatory molecule) were detected in 6/6 mares and 5/6 mares, respectively, from endometrial samples with tissue-adherent bacteria (*P* < 0.05). A greater incidence (*P* < 0.05) of Pel exopolysaccharide was present in samples fixed with Bouin's solution (18/18) than in buffered formalin (0/18), indicating that Bouin's solution is more appropriate for detecting bacteria adherent to the endometrium. There were no differences (*P* > 0.05) in the number of inflammatory cells in the endometrium between areas with and without tissue-adherent bacteria. Neutrophils were decreased (*P* < 0.05) in areas surrounding tissue-adherent bacteria compared to those in areas free of adherent bacteria. Gene expression of interleukin-10, an immune-modulatory cytokine, was significantly (*P* < 0.05) increased in areas of tissue-adherent bacteria compared to that in endometrium absent of biofilm. These findings indicate that P. aeruginosa produces a biofilm in the uterus and that the host immune response is modulated focally around areas with biofilm, but inflammation within the tissue is similar in areas with and without biofilm matrix. Future studies will focus on therapeutic options for elimination of bacterial biofilm in the equine uterus.

## INTRODUCTION

Bacterial endometritis that is refractory to traditional antimicrobial treatment is a significant challenge in the equine breeding industry (1–3). Production of biofilm is a common persistence strategy employed by bacterial pathogens for survival (4). Biofilms are complex and dynamic structured communities of bacteria that are resistant to clearance mediated by the host immune response and resistant to treatment with antimicrobial agents. The host immune system is often unable to recognize infections associated with biofilm due to the protective matrix of extracellular polymeric substances (EPS) surrounding the bacterial cells (5–10). The EPS matrix prevents antibodies from targeting bacteria within the biofilm and impedes white blood cell function and movement locally (11–14).

Growth in a biofilm protects bacteria from antibiotics by providing a diffusion barrier that decreases the effective concentration of antibiotics that can reach the protected bacterial colonies residing within the core of the biofilm, creating a nidus of infection (8, 15–18). Furthermore, the biofilm microenvironment slows down bacterial metabolism and therefore the replication rate of bacteria (9, 10, 19–22). As most antibiotics typically act only upon rapidly growing and dividing bacteria, the corresponding reduction in metabolic activity is associated with an increase in antimicrobial tolerance (6, 20–22).

In order for antimicrobial agents to penetrate into the biofilm, treatments must traverse through the EPS matrix, which is composed of exopolysaccharides, DNA, RNA, lipids, and proteins, in order to reach bacteria shielded within this protective barrier (23). P. aeruginosa produces three exopolysaccharides, alginate, Pel, and Psl (24–28). Alginate is a polymer consisting of β-1,4-linked l-guluronic and d-mannuronic acid; alginate alone is not sufficient for biofilm microcolony formation *in vitro* (29, 30). Psl is a pentasaccharide consisting of glucose, mannose, and rhamnose (25) that is involved in attachment of bacteria to a cellular or noncellular substrate (31, 32). Pel is composed of *N*-acetylgalactosamine and *N*-acetylglucosamine (33) and is responsible for attachment of the microcolony to a substrate and stabilization of extracellular DNA to provide support for the biofilm (33–35).

The bacterial cell signaling molecule cyclic di-GMP controls the switch from planktonic to biofilm phenotypes (36, 37). Two molecules of GTP are converted by diguanylate cyclases into cyclic di-GMP; conversely, phosphodiesterases break down cyclic di-GMP into pGpG and GMP (38–41). Cyclic di-GMP regulates the production of the exopolysaccharides alginate, Pel, and Psl *in vivo* and *in vitro* (42–44). The production of these exopolysaccharides is associated with development of antibiotic tolerance and increased resistance to the host immune response (45, 46).

The adaptation of antimicrobial tolerance and avoidance of the host immune system associated with bacteria growing in biofilm communities has created significant challenges in human medicine. The majority of hospital-acquired infections are associated with biofilm-forming bacteria (47), which increases treatment costs, exceeding a billion dollars annually (48–50). In equine medicine, studies evaluating biofilms in chronic infections are limited to a few pivotal studies. Comparisons of chronic nonhealing wounds on the distal equine limb revealed a significantly greater incidence of biofilm-producing bacteria near the wound site than from a skin sample from healthy tissue (51). Chronic uterine infections resistant to antimicrobial treatment may be due to biofilm production (52, 53). Recent work has shown that ∼80% of equine uterine isolates are capable of producing a biofilm *in vitro*, and clinical isolates of P. aeruginosa are capable of forming a biofilm *in vivo* (54–57).

The goal of this study was to determine the spatial localization of metabolically active bacteria in an equine model of biofilm-associated endometritis that allows for *ex vivo* bioluminescence imaging. Endometrial samples were analyzed for cyclic di-GMP levels, carbohydrate composition, histology, and immunohistochemistry to evaluate the association of the bacteria and characterize exopolysaccharide production during infection. Additionally, the host immune response was evaluated from samples of the cellular infiltrate in the endometrium and uterine lumen in order to measure host inflammatory gene expression.

## RESULTS

### Establishment of a uterine infection with P. aeruginosa.

None of the horses in this study exhibited signs of systemic illness following inoculation and prior to euthanasia, as evaluated by the following criteria and standards: no elevation in heart rate (abnormal, >36 beats per minute), respiratory rate (abnormal, >20 breaths per minutes), or body temperature (abnormal, >101.5°F). A discharge from the vulva was noted in one of the six mares (horse 2) on day 3 postinoculation.

**FIG 1:**
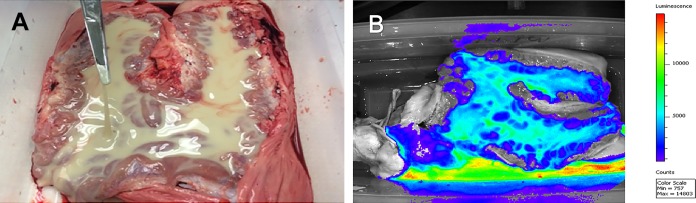
(A) Gross pathology of the equine endometrial surface of a representative mare 5 days postinoculation with *lux*-labeled P. aeruginosa. The uterine lumen was filled with 50 to 100 ml of purulent fluid. (B) Bioluminescent imaging of the equine uterus at 5 days postinoculation with *lux*-labeled P. aeruginosa. The highly luminescent areas are correlated with a high bacterial load of *lux*-expressing bacteria.

**FIG 2:**
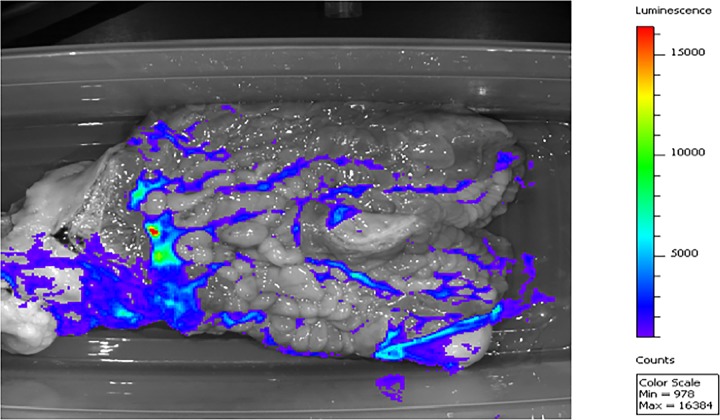
Bioluminescent imaging of the equine uterus at 5 days postinoculation with *lux*-labeled P. aeruginosa. Luminescence of a strongly adherent matrix on the endometrial surface was detected following repeated washing of the endometrium. The luminescence was present in the base of the uterus and extending into the uterine horns, indicating the presence of tissue-adherent *lux*-labeled P. aeruginosa.

Upon examination of the uterus, there was 50 to 100 ml of a tan, highly viscous, purulent fluid present in the uterine lumen (6 of 6 mares) (Fig. 1A). The fluid was highly luminescent, indicating a high bacterial load (Fig. 1B). After rinsing with Lactated Ringer's solution, the endometrium contained multifocal areas of luminescent tissue-adherent bacteria between the endometrial folds. The tissue-adherent bacteria extended from the base of and into both uterine horns (6/6 mares) (Fig. 2). Tissue-adherent bacteria were luminescent, indicating the presence of metabolically active *lux*-labeled bacteria within the adherent matrix. The luminescent signal was confirmed to be produced by *lux*-labeled P. aeruginosa during isolation and confirmation of P. aeruginosa isolates that were positive for luminescence in the samples collected from representative areas of the uterus.

The intraluminal fluid in the uterine lumen contained a heavy growth (too numerous to count) of *lux*-labeled P. aeruginosa, and no other bacterial species were isolated. The tissue-adherent bacteria had moderate growth (>30 colonies) of *lux*-labeled P. aeruginosa in all samples (6 of 6 mares). Sample collection from areas free of tissue-adherent bacteria had no growth in 3 of 6 mares and trace growth (<5 colonies) of *lux*-labeled P. aeruginosa from 3 of 6 mares. No other bacterial species (aerobic or anaerobic) were cultivated from any of the sampling sites with tissue-adherent bacteria or endometrium free of bacteria from any of the six mares. Using a model of equine endometritis, we could readily create an infection with P. aeruginosa clinical isolates in a repeatable fashion.

### P. aeruginosa produces a biofilm during equine endometritis.

Key molecules that are signatures of P. aeruginosa biofilm formation were analytically detected during the *in vivo* infection. The adherent EPS matrix contained a significantly greater incidence of the Pel exopolysaccharide (6 of 6 mares) compared to preinoculation endometrial samples (0 of 6 mares) (Table 1). The adherent material consisted predominantly of galactose, *N*-acetylgalactosamine, and *N*-acetylglucosamine (Table 1). The presence of *N*-acetylgalactosamine and *N*-acetylglucosamine, which are the major components of the Pel exopolysaccharide (33), provides evidence that this P. aeruginosa EPS matrix component contributes to biofilm formation in our equine endometritis model.

**TABLE 1:** Glycosyl composition analyses of samples from endometrium preinoculation and 5 days postinoculation with tissue-adherent bacteria

EPS component	Glycosyl residue^*a*^ (avg mol%)							
	Preinoculation (means ± SEM)	Postinoculation (means ± SEM)	Postinoculation for horse:					
			1	2	3	4	5	6
Fucose	5 ± 0.4^a^	2.3 ± 0.4^b^	1.6	2.2	3.4	3.3	1.2	2.3
Glucuronic acid	9 ± 0.6^a^	0.0 ± 0.0^b^	0	0	0	0	0	0
Galacturonic acid	11 ± 0.8^a^	0.0 ± 0.0^b^	0	0	0	0	0	0
Mannose	22 ± 1.2^a^	7.0 ± 2.0^b^	3.3	4.3	6.1	6.3	5.1	16.7
Galactose	22 ± 1.8^a^	36.3 ± 1.3^b^	41.1	31.9	38.3	36.3	33.7	36.7
Glucose	18 ± 1.3^a^	3.3 ± 0.3^b^	3.1	2.8	2.8	4.1	4.5	2.5
*N*-Acetylgalactosamine	8 ± 0.7^a^	23.4 ± 2.5^b^	18.7	30.5	19.3	22.9	31.3	17.7
*N*-Acetylglucosamine	5 ± 0.1^a^	27.7 ± 1.3^b^	32.2	28.3	30.2	27.1	24.2	24.2

a A difference in superscript letter indicates a significant difference (*P* < 0.05). A significant difference (*P* < 0.05) in the glycosyl composition distribution was present between the pre- and postinoculation samples. After inoculation, a significantly greater amount of *N*-acetylgalactosamine and *N*-acetylglucosamine was detected (6 of 6 mares) compared to that in preinoculation samples. *N*-Acetylgalactosamine and *N*-acetylglucosamine are the main components of the Pel exopolysaccharide, which is a known matrix component of P. aeruginosa biofilm. Glycosyl residues (ribose, arabinose, rhamnose, and xylose) that were below the limit of detection in the samples are not represented.

**FIG 3:**
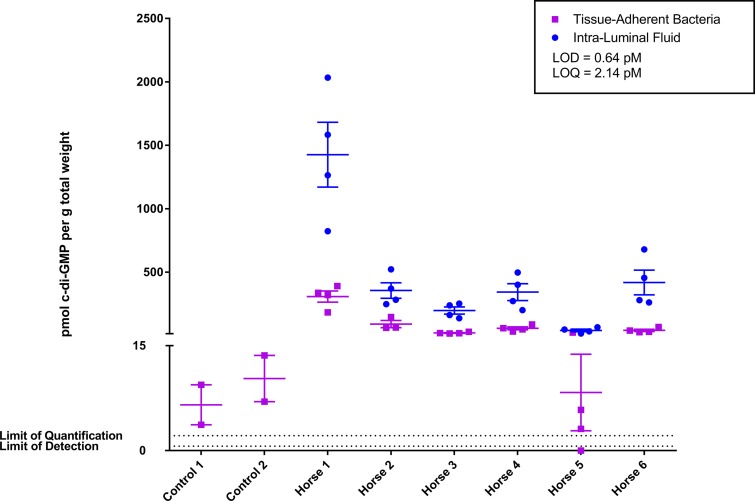
LC-MS/MS quantitative analysis of the bacterial secondary messenger molecule, cyclic di-GMP, from uterine infections to detect P. aeruginosa biofilms. Elevated cyclic di-GMP levels were detected in a majority of tissue-adherent bacterial samples, except for those from samples obtained from horse 5, which were not elevated compared to those of control samples. Four samples of intraluminal fluid and tissue-adherent bacteria were collected at random locations from each infected uterus (*n* = 6). Four control samples were collected by uterine biopsy procedure from two uninfected mares. Amounts are represented as picomoles of cyclic di-GMP per gram of sample. The calculated limit of detection (LOD) is 0.64 pmol, and the calculated limit of quantification (LOQ) is 2.14 pmol. Experimental samples range from 3.1 pmol/g to 2,033 pmol/g cyclic di-GMP. Samples from which no cyclic di-GMP was detected are represented under the LOD line.

Additionally, the intraluminal fluid in the uterus and the tissue-adherent bacteria contained detectable levels of cyclic di-GMP, a cell-signaling molecule that promotes biofilm formation in bacteria and is not produced by mammals (Fig. 3). A significantly (*P* < 0.05) greater amount of cyclic di-GMP was detected in all six samples of intraluminal fluid compared to that from tissue samples from uninoculated horses free of infection. Tissue-adherent bacteria had significantly (*P* < 0.05) elevated levels of cyclic di-GMP in five of six horses compared to those of uterine endometrium from horses free of adherent bacteria. There was significantly greater (*P* < 0.05) mean levels of cyclic di-GMP in the intraluminal fluid (463.7 ± 102.7 pmol/g) than in the tissue-adherent bacterial samples (87.1 ± 23.61 pmol/g). It should be noted that the tissue-adherent bacterial samples have a greater abundance of host tissue than bacterial cells, and the intraluminal fluid samples have a large amount of bacteria with very few host cells. The samples were normalized by weight for comparison, and it would be expected that the samples with a greater amount of host tissue would have a reduced concentration of cyclic di-GMP detected. These results indicate that key P. aeruginosa biofilm signatures, which include cyclic di-GMP and the Pel exopolysaccharide, were detectable in the tissue-adherent bacteria from equine endometritis samples.

### Microscopic evaluation of tissue-adherent biofilm.

Histologic evaluation (hematoxylin and eosin [H&E] staining) of endometrial tissue samples was performed by an independent pathologist without knowledge of the study design. The inflammatory response within the endometrial tissue was classified as a severe, diffuse, lymphocytic infiltrate that was not different from samples with tissue-adherent bacteria (6 of 6 mares) and samples absent of bacteria (6 of 6 mares). There was no significant difference in the inflammatory response detected in the endometrium for samples collected in the uterine body and the uterine horn, as severe inflammation was observed at both sites in all mares. These findings indicate that the inflammatory response was similar throughout the endometrial tissue of the uterus irrespective of the presence or absence of tissue-adherent bacteria at specific focal sites of infection.

**FIG 4:**
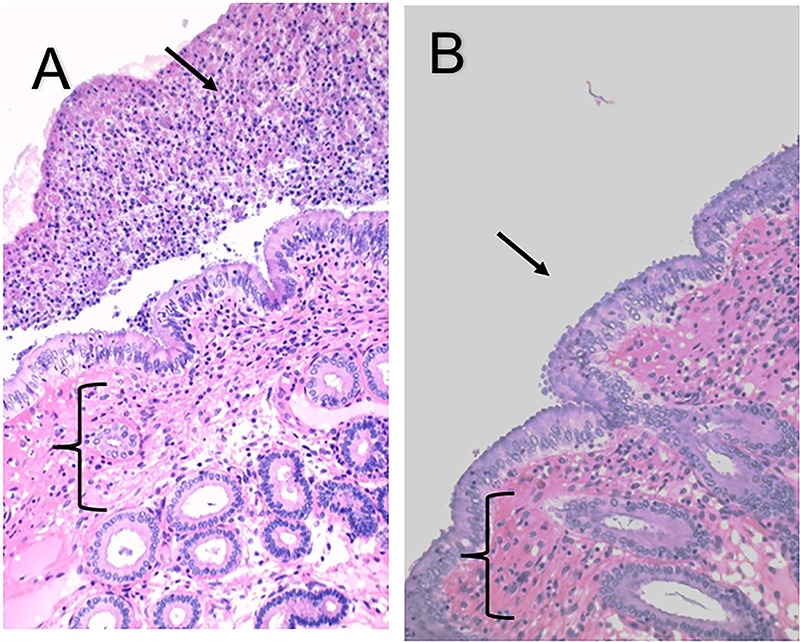
Detection of tissue-adherent P. aeruginosa from the equine endometrium was dependent on the fixative. H&E-stained endometrial sections from a representative mare with tissue-adherent P. aeruginosa are shown. The inflammatory response (brackets) in the endometrium was a severe lymphocytic infiltrate. (A) Tissue was fixed in Bouin's solution, and the tissue-adherent P. aeruginosa was clearly evident (black arrow). (B) Tissue was collected from the same mare and same location as those described for panel A but was fixed in 10% formalin. Note the lack of tissue-adherent P. aeruginosa in panel B. The black arrow points to where the tissue-adherent P. aeruginosa should be located.

**FIG 5:**
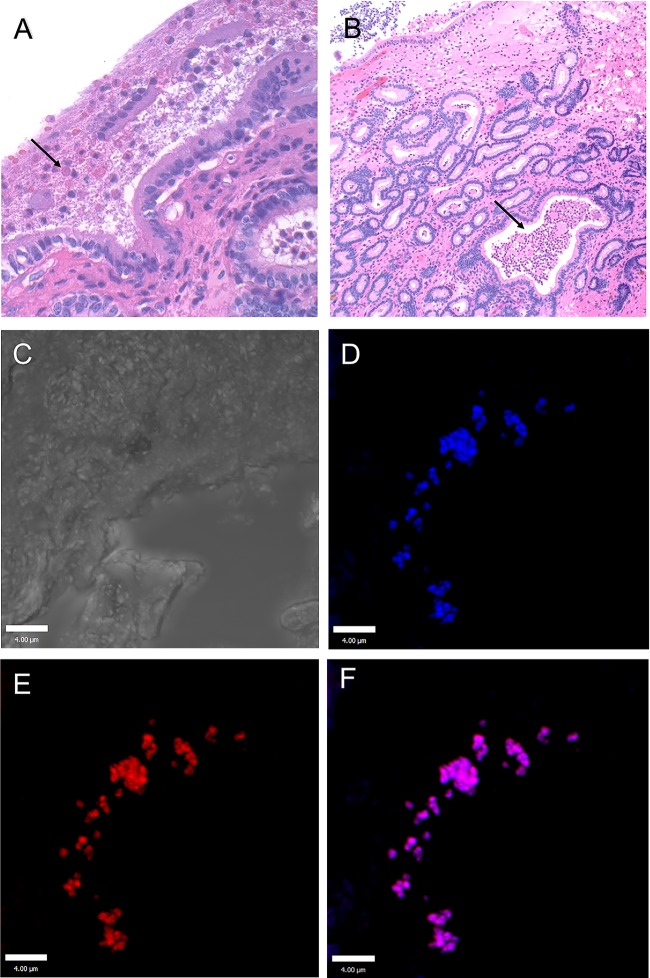
Detection of tissue-adherent P. aeruginosa in endometrium samples. H&E image of endometrium with tissue-adherent P. aeruginosa on the luminal surface (black arrow) (A) and deep in the endometrial glands (black arrow) (B). (C) Differential interference contrast image of an endometrial gland below the luminal surface of the uterus; this is similar to the area represented in panel B by the black arrow. Immunofluorescent staining of tissue-adherent P. aeruginosa with an anti-Pseudomonas antibody (Alexa Fluor 405) (D) and anti-Pel lectin (Texas red) (E) and merged image detecting the Pel exopolysaccharide colocalized with P. aeruginosa (F). Immunofluorescent images are projected images of Z-stacks as processed by Volocity image analysis software in which 0.5-μm scanning increments were performed through approximately 10 μm of tissue. The scale bar is 4 μm.

Tissue-adherent bacteria were detected in a significantly greater number of samples fixed in Bouin's solution (18 of 18 samples) than in 10% formalin (0 of 18 samples) following routine histologic processing (Fig. 4A and B). Histologic evaluation of the tissue-adherent bacteria revealed that the tissue-adherent bacteria contained host epithelial cells, white blood cells, bacteria, and other nonidentifiable constituents (Fig. 4A). The tissue-adherent bacteria were present on the luminal surface of the endometrium and deeper within the tissue of the endometrial glands (Fig. 5A and B). The glands of the endometrium are responsible for producing a fluid rich in protein, carbohydrates, and lipids in order to support the developing conceptus. The bacteria potentially were localized deeper in the glands to access the nutrient-rich glandular fluid.

Adjacent tissue sections were evaluated by immunohistochemistry (IHC) for the presence of P. aeruginosa and Pel exopolysaccharide. Localization of both P. aeruginosa and Pel was observed in the samples with tissue-adherent bacteria using confocal microscopy. The greatest abundances of bacteria and Pel were present in the uterine glands (Fig. 5C to E). Fluorophores conjugated to an antibody and a lectin specific to P. aeruginosa and Pel could be colocalized, indicating that P. aeruginosa and the Pel exopolysaccharide were intimately associated during the infection (Fig. 5F). There was also significantly greater incidence of simultaneous detection and colocalization of the bacteria and the associated Pel exopolysaccharide in samples from tissue-adherent bacteria (6/6 mares) than from samples from areas with no adherent bacteria (0/6 mares).

### Characterization of the host immune response.

Cytological evaluation of the intraluminal fluid revealed severe inflammation (>5 neutrophils per 400× field of view, 6 of 6 mares), with a predominant neutrophil-driven immune response. Both aggregating and nonaggregating rod-shaped bacteria could be visualized. After rinsing, endometrial cytology samples collected from tissue adjacent to the adherent bacteria had significantly fewer neutrophils (*P* < 0.05) (0 neutrophils per high-power field [HPF], 6 of 6 mares) than samples collected from areas with no bacteria present (>5 neutrophils per HPF, 6 of 6 mares). The decrease in neutrophils on the endometrial surface surrounding the tissue-adherent bacteria suggests that, at least locally within the uterine lumen, the cellular host immune response was modulated.

**FIG 6:**
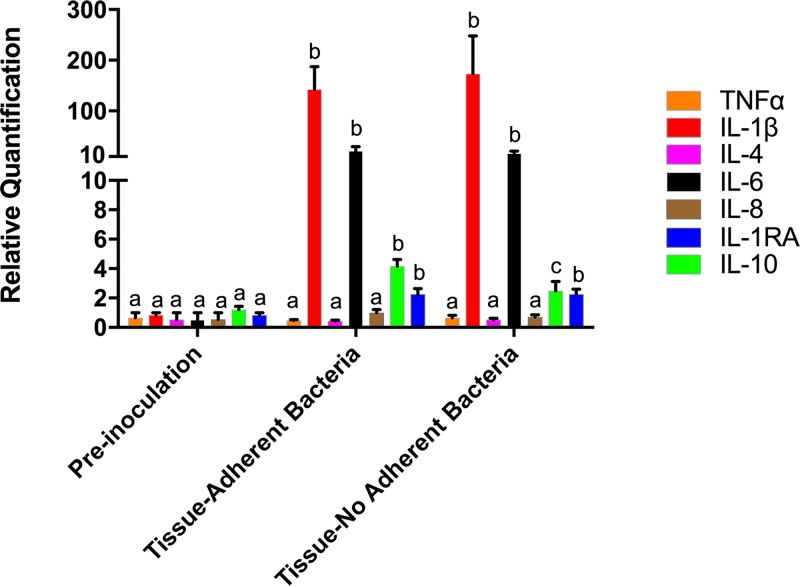
Fold change in gene expression of inflammatory cytokines in the endometrium preinoculation, postinoculation with tissue-adherent P. aeruginosa, and postinoculation free of tissue-adherent P. aeruginosa. A proinflammatory response was noted with upregulation of IL-6 and IL-1β. Endometrium with tissue-adherent P. aeruginosa had significantly greater change in gene expression of IL-10, an immune-modulatory cytokine, than endometrium free of bacteria. A difference in lowercase letter indicates a significant difference in gene expression (*P* < 0.05).

Cytokine gene expression (tumor necrosis factor alpha [TNF-α], interleukin-1β [IL-1β], IL-4, IL-6, IL-8, IL-10, and IL-1-RA) was monitored in the endometrium to determine the immune response in the uterine lumen. As expected, a significant increase (*P* < 0.05) in the expression of cytokines was detected in samples between preinoculation and postinoculation (Fig. 6). Genes expressing the proinflammatory cytokines IL-1β and IL-6 had the greatest increase in expression between the preinoculation and postinoculation samples (Fig. 6). IL-1β and IL-6 both stimulate the proinflammatory response during infection, resulting in severe inflammation, and are known to be upregulated following inoculation with bacteria (58). IL-10 was the only cytokine that was expressed with significant difference (*P* < 0.05) in postinoculation samples when comparing endometrium with tissue-adherent bacteria and endometrium that did not have adherent bacteria (Fig. 6). IL-10 is an immune-modulatory cytokine that inhibits the secretion of inflammatory cytokines, including IL-1β (59, 60). Increased production of IL-10 in the endometrium with tissue-adherent bacteria may partially explain the decrease in the number of neutrophils present on the endometrial surface near areas with tissue-adherent bacteria.

## DISCUSSION

The link between biofilms and chronic infections is well recognized in dentistry and human medicine (61–63). In veterinary medicine, this association is not as well defined, but the clinical opinion is that chronic infections often involve a biofilm potentially leading to increased morbidity and mortality (51, 64, 65). Bacterial isolates from the equine uterus are capable of forming a biofilm *in vitro*, similar to disease-causing bacterial isolates from human and veterinary medicine (56).

Biofilms are aggregates of bacteria surrounded by an extracellular polymeric substance produced by the bacteria (16). Numerous chronic infections have been recognized to be associated with biofilms in naturally occurring infections and animal models (13, 66–68). The current study identified and localized P. aeruginosa biofilms on the equine endometrial surface and also deeper in the tissue within endometrial glands. These findings are supported by the detection of cyclic di-GMP, which is an intracellular signaling molecule that initiates and maintains the biofilm phenotype of bacteria. Furthermore, detection of the Pel exopolysaccharide, a key EPS matrix component produced in P. aeruginosa biofilm, provides additional evidence in support of biofilm production during P. aeruginosa infection. These findings specifically support the link between biofilms and chronic infections in equine reproduction.

The EPS matrix of the bacterial biofilm protects the bacteria from eradication by the host immune system, resulting in development of a chronic infection. The biofilm lifestyle is recognized to produce less of a host immune response than bacteria in a planktonic state (5–9, 11, 12). Biofilm-associated infections also result in reduced oxidative function and phagocytosis of bacteria, which prevents the host immune cells from actively clearing the infection (11, 69, 70). Additionally, the biofilm phenotype prevents the recognition of an active infection in the host by reducing the proinflammatory responses (11–13, 63, 64).

In the current study, we noted that the amount of inflammation and expression of proinflammatory cytokine genes within the endometrial tissue was similar regardless of the presence or absence of tissue-adherent bacteria on the endometrial surface. These findings may be due to stimulation of the host immune system by the intraluminal fluid containing bacteria in a planktonic and biofilm state. Previously published research has also suggested that equine uterine infections are focal in nature, even though the uterine inflammatory response is diffuse and not confined specifically to the site of focal infection (71). Clinically this suggests that if unexplainable inflammation is present during an endometrial biopsy procedure, a focal infection could be present in an area not adjacent to the site of biopsy sample collection.

The local cellular host immune response was modulated with a reduction in neutrophils surrounding the tissue-adherent bacteria on the endometrial surface compared to areas free of tissue-adherent bacteria. Elucidation of the mechanism of action for altering host immune response was not possible in this study, as only a minor increase in IL-10 gene expression was observed in areas with tissue-adherent bacteria. An alternative cause of the reduced neutrophils near adherent bacteria is the extensive washing that was performed on the sites, possibly resulting in removal of a greater number of neutrophils than from areas without adherent bacteria. Studies of the equine uterus are difficult, as few validated reagents exist for evaluating the host inflammatory response and the majority of human reagents (such as antibodies) do not function in assays that monitor the horse. In addition, the inability to observe a change in inflammatory mediators may be due to the presence of bacteria in both biofilm and planktonic states throughout the uterus. Further research is warranted to determine the mechanism of action to explain the reduced neutrophil population near areas with adherent bacteria.

Diagnostics for detecting a broad spectrum of *in vivo* biofilms are limited in application due to the variation between bacterium-specific exopolysaccharides that are signatures of a biofilm infection. The universal second messenger, cyclic di-GMP, is a well-known bacterial signaling molecule that regulates biofilm formation and controls the shift from a planktonic to biofilm phenotype (36, 37, 72–75). Elevated levels of cyclic di-GMP *in vitro* are associated with an increased production of EPS matrix components that are linked to biofilm-associated infections (45, 72, 76, 77). In the current study, cyclic di-GMP levels were detected in the intraluminal uterine fluid and in tissue with adherent bacteria during an active infection. However, cyclic di-GMP was not detected in all mares or samples. In one mare (number 5), cyclic di-GMP was relatively undetectable in tissue-adherent bacteria compared to tissue from uninoculated mares, even though adherent bacteria and the Pel polysaccharide could be detected by other means. Future research will address if this is the result of intrinsic factors associated with the individual animal, if collection of the samples occurred before a significant rise in cyclic di-GMP in the cases where cyclic di-GMP was undetected, or if cyclic di-GMP was degraded during sample preparation for analysis.

This is the first study to utilize cyclic di-GMP as a marker of biofilm-associated infection *in vivo*. The majority of samples analyzed after inoculation were positive for cyclic di-GMP. In normal mares that were demonstrated to be free of infection based on clinical history, endometrial microbial culture, and cytological evaluation, cyclic di-GMP was detectable at very low levels (8.5 ± 2.1 pM). Detection of cyclic di-GMP in normal equine endometrium could be due to bacterial contamination during sample collection or the presence of a subclinical infection in the control mares classified as being free of infection. To collect the control biopsy samples, the uterus is accessed through the vulva and vaginal vault, which presents an inherent risk of contamination from the normal bacterial flora in the vagina. The uterus of the mare is often considered a privileged site that does not have a normal flora (78). However, the uterus is constantly being exposed to bacteria ascending through the cervix and vagina (78–80). The low level of cyclic di-GMP detected in the control mares could be from subclinical bacterial exposure that is not significant enough to be detected with routine microbial culture and cytology screening methods. The use of cyclic di-GMP as a metabolic signature of active bacterial biofilm production has the potential for use as a diagnostic marker to identify biofilm-associated infections *in vivo*. Future research is required to develop a fast and sensitive assay to detect cyclic di-GMP as a biomarker of biofilm-associated infections and for improved characterization of the endometrium in normal, subclinical, and clinical situations.

In conclusion, clinical isolates of P. aeruginosa from the equine uterus can produce a biofilm *in vivo* in an established model of bacterial endometritis. The presence of biofilm matrix during this infection was confirmed using detection of cyclic di-GMP, immunofluorescence of bacteria and exopolysaccharides, and identification bacterial exopolysaccharides by carbohydrate analysis. The corresponding inflammatory response within the uterine lumen is also altered in foci containing bacteria that are producing a biofilm. Interestingly, the inflammation observed deeper within the endometrial tissue was similar between areas involving a bacterial biofilm and areas without a bacterial biofilm. Future efforts will be directed toward understanding how these infections develop and persist when challenged by the host immune system and the effects of various therapeutic treatments that target biofilm-associated infections. This knowledge will provide the veterinary community with improved diagnostics and therapeutics to identify and treat bacterial biofilm-associated infections.

## MATERIALS AND METHODS

### Imaging and localization of the biofilm in the equine uterus.

An established model of infectious endometritis was used in which mares (*n* = 6) were treated with a 200-mg intramuscular injection of natural progesterone in cottonseed oil daily for 5 days prior to inoculation (81). The mares used in this study were confirmed to be free of infection by sampling for microbial growth from endometrial swabs and evaluating the uterine lumen for inflammatory cells 72 h prior to the first injection of progesterone. A biopsy sample of the endometrium was collected 7 to 10 days prior to the first progesterone injection and was free of inflammatory cells. After 5 days of progesterone treatment, the uterus was inoculated with a mixture of three previously published *lux*-labeled P. aeruginosa isolates (PA004, PA035, and PA069) modified to constitutively express the luminescent reporter genes *luxCDABE* (56). This method was validated previously to ensure the establishment of a biofilm-associated infection (56). Overnight cultures of the three *lux*-labeled isolates were diluted to a final concentration of 1 × 10^6^ bacteria in a final volume of 3 ml of 1× phosphate-buffered saline (PBS) for inoculation as previously described (56). The mares were euthanized 5 days after inoculation. The reproductive tract was removed by cutting open the entire endometrial surface (including the area between endometrial folds). Bacterial luminescence was quantified/visualized with the IVIS 200 imaging system (PerkinElmer, Waltham, MA). Focal, random areas of luminescence, which indicate the presence of metabolically active bacteria, were rinsed three times with 240 ml (∼750 ml total) of Lactated Ringer's solution pressurized through a 20-gauge hypodermic needle. The high-pressure irrigation achieved with this method is able to effectively remove bacteria from tissue (82–85). The explanted uterus was evaluated for luminescence a second time after the stringent rinsing to further quantify the luminescence of bacteria adherent to the endometrial surface. This study was approved by Colorado State University's Institutional Animal Care and Use Committee and Institutional Biosafety Committee.

### Aerobic and anaerobic culture.

Culture swabs were placed within the intraluminal fluid in the uterine lumen (prerinsing), the tissue-adherent bacteria (postrinsing), and in areas without bioluminescent bacteria for 15 s. Sampling sites were determined based on the amount of bioluminescent signal. The swabs were streaked onto LB agar for aerobic growth at 37°C. Bacterial growth was evaluated by an IVIS imager for luminescence to determine if the colonies were from the original inoculation. If colonies were present that were not luminescent, they were submitted for identification at the Colorado State Veterinary Diagnostic Laboratory. A swab from each sample site was also submitted to the Colorado State Veterinary Diagnostic Laboratory for cultivation of anaerobic bacteria.

### IHC and lectin staining.

Endometrium with luminescence-positive tissue-adherent bacteria and endometrium free of adherent bacteria (samples without luminescence) were processed for immunohistochemistry (IHC) to potentially localize bacteria and associated biofilm EPS. Samples were randomly allocated for fixation in 10% formalin prior to embedding or fixed in Bouin's solution for 12 to 18 h, followed by 70% ethanol in preparation for paraffin embedding. Paraffin-embedded tissue was sectioned from the tissue block in 10-μm sections. Slides were deparaffinized using an ethanol gradient, and nonspecific staining was blocked using BioCare Sniper block (BioCare Inc., Concord CA). The slides were incubated with a primary 1:800 anti-Pseudomonas rabbit antibody, PA1-73116, raised against whole cells of P. aeruginosa ATCC 27853 (Thermo Fisher Scientific, Rockford IL), a secondary 1:500 biotin-conjugated donkey anti-rabbit antibody (Jackson ImmunoResearch Laboratories, West Grove, PA), and lastly a 1:500 streptavidin-conjugated Alexa Fluor 405 probe (Thermo Fisher Scientific, Rockford, IL). An anti-Pel Texas red-conjugated Wisteria floribunda lectin (EY Labs, San Mateo, CA) was applied for colocalization of P. aeruginosa and the Pel exopolysaccharide. Localization of bacteria was visualized using an Olympus IX81 inverted confocal microscope. Images were analyzed with Volocity image analysis software (PerkinElmer, Waltham, MA).

### Carbohydrate analysis of the biofilm EPS.

Glycosyl composition analysis was conducted at the University of Georgia's Complex Carbohydrate Research Center on endometrium prior to inoculation and endometrium postinoculation containing tissue-adherent bacteria. Samples were analyzed by combined gas chromatography-mass spectrometry (GC-MS) of the per-O-trimethylsilyl derivatives of the monosaccharide methyl glycosides produced from the sample by acidic methanolysis, as described previously by Santander et al. (86).

### Cyclic di-GMP extraction.

Four samples of intraluminal fluid and tissue-adherent bacteria were collected at random locations from each infected uterus (*n* = 6). Four control samples were collected by a uterine biopsy procedure from two uninfected mares. Nucleotide extraction methods using perchloric acid were adapted from Irie and Parsek (87). Tissue samples with adherent bacteria and intraluminal fluid were collected in quadruplicate from randomly selected locations of each uterus. Biological samples were maintained at 37°C during collection and transport between laboratories. The wet weight of each sample was recorded prior to the extraction procedure for normalization. Samples were suspended in 900 μl fresh chilled extraction buffer (80% liquid chromatography-MS [LC-MS]-grade water and 20% acetonitrile) containing an internal standard, 2-chloroadenosine-5′-O-monophosphate (2-Cl-5′-AMP; Axxora, LLC, Farmingdale, NY), at 100 nM. Perchloric acid (70%, vol/vol) was added to a final concentration of 0.6 M, and each sample was vortexed for 5 s, followed by a 30-min incubation on ice. Samples were spun at 16,000 × *g* for 10 min at 4°C. Supernatant was transferred to a larger 15-ml conical tube before the addition of 219 μl 2.5 M KHCO_3_ for acid neutralization. Neutralized samples were spun at 4,000 × *g* for 10 min at 4°C before the supernatant was removed and transferred to new 1.7-ml tubes. Samples were spun at 16,000 × *g* for 10 min at 4°C to remove remaining perchlorate salt precipitates. A calibration curve using known standards of chemically synthesized c-di-GMP in a 3-fold dilution series was generated in fresh chilled extraction buffer (80% LC-MS-grade water and 20% acetonitrile) containing 100 nM 2-Cl-5′-AMP.

### Cyclic di-GMP quantification.

LC-tandem MS was performed on a Waters Acquity M-class ultraperformance liquid chromatograph coupled to a Waters Xevo TQ-S triple-quadrupole mass spectrometer. Chromatographic separations were carried out on a Waters Atlantis dC_18_ stationary-phase column (300 μm by 150 mm, 3.0-μm diameter). Mobile phases were 99.9% acetonitrile, 0.1% formic acid (B), and water with 0.1% formic acid (A). The following analytical gradient was used: time of 0 min, 5% B; time of 6.0 min, 97% B; time of 7 min, 97% B; time of 8 min, 5% B; time of 13 min, 5% B. The flow rate was 11.5 μl/min, and the injection volume was 1.0 μl. Samples were held at 4°C in the autosampler, and the column was operated at 30°C. The MS was operated in selected reaction monitoring (SRM) mode. Transition ion mass-to-charge ratios (*m/z*) were the following for cyclic di-GMP: 691.1 > 540.1, 691.1 > 248.1, and 691.1 > 152.1. Transition ions for the internal standard (2-chloro-AMP) were set at 382.0 > 170.1. Product ions, collision energies, and cone voltages were optimized for each analyte by direct injection of individual synthetic standards. Interchannel delay was set to 3 ms. The MS was operated in positive ionization mode with the capillary voltage set to 3.6 kV. Source temperature was 120°C, and desolvation temperature was 350°C. Desolvation gas flow was 1,000 liters/h, cone gas flow was 150 liters/h, and collision gas flow was 0.2 ml/min. Nebulizer pressure (nitrogen) was set to 7 × 10^5^ Pa. Argon was used as the collision gas. A calibration curve was generated using authentic standards for each compound and their corresponding stable isotope-labeled internal standards in 100% methanol solution.

### LC-MS data analysis and statistics.

All raw data files were imported into the Skyline open-source software package (88). Each target analyte was visually inspected for retention time and peak area integration. Peak areas were extracted for target compounds detected in biological samples and normalized to the peak area of the appropriate internal standard in each sample. Normalized peak areas were exported to Excel, and absolute quantitation was obtained by using the linear regression equation generated for each compound from the calibration curve. Limits of detection (LOD) and limits of quantification (LOQ) were calculated as 3 times or 10 times, respectively, the standard deviations from the blank divided by the slope of the calibration curve (89, 90). The LOD of cyclic di-GMP was determined to be 0.64 pM with an LOQ of 2.14 pM.

### Endometrial cytology.

A cytology brush was gently rolled in contact with the endometrium for 15 s in areas with intraluminal fluid in the uterine lumen (prerinsing), areas with tissue-adherent bacteria (after rinsing), and areas with no bacteria (after rinsing) based on luminescence. The brush was gently rolled onto a glass slide, allowed to air dry, and stained with a modified Wright's stain. Slides were assessed by a nonbiased evaluator for the presence, quantity, and characterization of inflammatory cells (91). The average numbers of white blood cells per high-power field (HPF) (400× magnification) were determined using a standardized scoring system (91).

### Histology.

Tissue sections from the paraffin blocks described above were stained with hematoxylin and eosin (H&E) stain. This slide was evaluated by a nonbiased board-certified veterinary pathologist for the degree and population of inflammatory cells in five areas of the endometrium.

### Inflammatory cytokine expression.

Biopsy samples of tissue-adherent bacteria and a sample free of adherent bacteria were snap-frozen for analysis of gene expression and were taken following the rinsing of the endometrium. In order to evaluate gene expression by quantitative reverse transcription-PCR, RNA was extracted using the Qiagen RNeasy minikit (Qiagen Sciences, Germantown, MD) according to the manufacturer's instructions with optional DNase treatment. Subsequently, 1 μg total RNA was used as the template to synthesize cDNA with the high-capacity cDNA reverse transcription kit (Applied Biosystems, Foster City, CA). Primers were designed using Primer3 (92), and melting curve analysis was performed to ensure single-product amplification for all primer pairs. Real-time PCR was performed on an ABI 7900HT fast real-time PCR system (Applied Biosystems) using previously validated assays specific for each equine gene of interest (IL-1RA, IL-1, IL-4, IL-6, IL-8, IL-10, and TNF-α) (93). Each reaction well contained 5 μl of SYBR green PCR master mix, cDNA equivalent to 20 ng of total RNA, and 400 nM (each) forward and reverse amplification primers. Cycling conditions were 95°C for 10 min followed by 40 cycles of 95°C for 15 s and 60°C for 1 min. The experimental cycle threshold was calibrated against the reference gene β-actin, which is constitutively expressed across the estrous cycle (94, 95). Samples were analyzed for relative gene expression by the ΔΔ*C_T_* method (where *C_T_* is threshold cycle) (96).

### Statistical analysis.

Comparisons were performed by one-way analysis of variance by applying the Tukey-Kramer multiple-comparison test using SAS. Treatment interactions and inflammation in the endometrium were evaluated with a mixed-model analysis of variance. Fixed variables were the collection periods (prior to inoculation or postinoculation). Both main and interaction effects were assessed, where the horse is considered a random effect. Categorical data were compared using chi-square analysis. Data are presented as the means ± standard errors of the means (SEM).
